# Conceptualisations of curriculum co-creation: ‘it’s not them and us, it’s just *us*’

**DOI:** 10.1007/s41297-022-00180-w

**Published:** 2022-12-28

**Authors:** Tanya Lubicz-Nawrocka

**Affiliations:** The University of Stirling, Stirling, Scotland

**Keywords:** Curriculum co-creation, Curriculum negotiation, Student engagement, Higher education

## Abstract

Building on the growing field of curriculum co-creation in higher education, this research analyses current conceptualisations of this concept based on the perceptions of student and staff co-creation practitioners. It draws on a rigorous review of international curriculum co-creation literature and describes in-depth research on experiences and conceptualisations of fifteen curriculum co-creation initiatives across eight subject areas at five universities in Scotland. Following an inductive analysis, the findings highlight conceptualisations of curriculum co-creation that focus on (A) developing shared values, (B) enhancing creativity through collaboration and (C) negotiating power for mutual benefit. These findings are discussed with respect to how they contribute to a new definition: *curriculum co-creation is a relational way of working underpinned by shared responsibility, reciprocity in learning from each other, mutual respect, care, trust and empathy. This values-based, creative process helps staff and students work together to share and negotiate decision-making about aspects of curricula, which often leads to mutual benefits for learners and teachers.* This new definition encompasses current conceptualisations of curriculum co-creation in the rapidly-changing higher education sector, with implications for fostering resilient, authentic and meaningful pedagogies that are relevant to today’s challenges in higher education and beyond.

## Introduction

Engagement of higher education students and staff is an increasing concern, especially since the COVID-19 pandemic when many staff and students are now balancing online and in-person learning and teaching with many other responsibilities. One way of addressing this is to find ways to advance engaging, meaningful and authentic forms of learning and teaching such as curriculum co-creation. In the last decade, there has been a dramatic increase in the discourse on curriculum co-creation (Bovill & Woolmer, 2019) and wider students-as-partners initiatives (Healey & Healey, 2018; Mercer-Mapstone et al., 2017). Many terms are often overlapping, including working with students as partners through co-creation/co-design/co-production both *of* and *in* the curriculum and/or learning and teaching (Bovill & Woolmer, 2019; Mercer-Mapstone et al., 2017). Although the burgeoning literature has been beneficial, there is a lack of clarity about what curriculum co-creation can be, beyond staff and students’ active engagement and collaborations in learning and teaching.

This paper acknowledges the overlaps between curriculum co-creation and partnership research, and it focuses on the under-problematised theory and practice of curriculum co-creation. By taking a critical perspective of the literature with respect to a recent study of co-creation examples across the Scottish higher education context, this research contributes to the existing body of knowledge by reconsidering definitions of co-creation and adding new perspectives for adopting it in practice. It also explores implications for advancing authentic and meaningful curricula that address the challenges we face in today’s quickly changing world.

Whilst there is a wide literature on school curricular development and planning, the term is rarely defined in the higher education context and, even when it is, there are many different interpretations (Fraser & Bosanquet, 2006; Lattuca & Stark, 2009). Drawing on Fraser and Bosanquet’s work (2006), I acknowledge the many conceptualisations of higher education curricula, ranging from product-focused, teacher-directed stances to process-focused, student-centred views. How different individuals view the curriculum influences the possibilities that they envision for curriculum co-creation opportunities (Bovill & Woolmer, 2019). Boomer’s work has been particularly generative by suggesting a ‘curriculum-as-negotiation’, bringing together teacher-centred and student-centred curriculum design whilst emphasising a process-focused view of the curriculum as a verb, ‘curriculuming’ (Boomer, 1992; Green, 2021). Boomer (1992, p. 14) wrote:

Negotiating the curriculum means deliberately planning to invite students to contribute to, and to modify, the educational programme, so that they will have a real investment both in the learning journey and in the outcomes. Negotiating also means making explicit, and then confronting, the constraints of the learning context, and the non-negotiable requirements that apply.

Boomer’s work has also been influential in acknowledging democratic values and issues of power (Green, 2021) and by setting the scene for more recent literature curriculum studies including those in curriculum co-creation (Breen & Littlejohn, 2000; Bron et al., 2016; Green, 2021). Inspired by the work of Boomer (1992), Fraser and Bosanquet (2006) and Barnett and Coate (2004) in particular, I see the curriculum as a creative, student-centred space where staff and students engage in a process of learning and teaching that they continually adapt—and can take up opportunities to co-create within certain constraints—to meet their shared objectives of developing students’ knowledge, skills and wider capabilities.

## Existing definitions of curriculum co-creation

Two of the most widely cited definitions of curriculum co-creation focus on collaboration and partnership between students and staff in curriculum development activities. Bovill et al., (2011, p. 137) suggest that ‘co-creation of curricula implies students and academic staff working in partnership to create some or all aspects of the planning, implementation and evaluation of the learning experience’. Ryan and Tilbury’s (2013, p. 16) Higher Education Academy report drew on this definition, focusing on knowledge co-creation:

The concept of ‘co-creation’ is used to indicate interactions that encourage collaborative and democratic input from students as stakeholders in shaping knowledge practices…. The pedagogical ambitions behind learner empowerment are realised through the use of participatory, transformative and ‘active’ pedagogies...

Subsequent definitions also emphasise collaboration; for example, a widely cited definition by Bovill et al., (2016, p. 196) states ‘co-creation of learning and teaching occurs when staff and students work collaboratively with one another to create components of curricula and/or pedagogical approaches’. A subsequent definition by Dollinger et al., (2018, p. 210) draws on marketing: ‘value co-creation is the process of students’ feedback, opinions and other resources such as their intellectual capabilities and personalities, integrated alongside institutional resources, which can offer mutual value to both students and institutions’.

Definitions of curriculum co-creation often draw on the concept of partnership, with the latter having emerged as the predominant term in the literature encompassing a wide range of curricular and extracurricular initiatives (Cook-Sather et al., 2018b; Mercer-Mapstone et al., 2017). There are many synergies between curriculum co-creation and the concept of pedagogical partnership. This can be seen in how Cook-Sather, Felten, and Bovill—who individually and in collaboration are extremely influential in this field—used the term co-creation when publishing in 2011 and 2016 but referred to partnership in their pivotal 2014 book. Their widely cited definition of student/staff partnership is also important and of relevance: ‘a collaborative, reciprocal process through which all participants have the opportunity to contribute equally, although not necessarily in the same ways, to curricular or pedagogical conceptualization, decision-making, implementation, investigation or analysis’ (Cook-Sather et al., 2014, pp. 6–7). These authors also describe the important values of respect, reciprocity and shared responsibility. Although both partnership and co-creation initiatives often seek to include diverse student voices whilst enhancing learning and teaching (Cook-Sather et al., 2018a; Lubicz-Nawrocka, 2019a), many partnership examples involve small numbers of already highly engaged students in optional projects in contrast to whole-class co-creation initiatives that are embedded into coursework (Bovill, 2020b). Bovill (2020a) suggested that co-creation and partnership often overlap in their emphasis on democratic values and shared decision-making, although partnership can imply a level of equality that some staff find hard to align with the reality of academic structures and constraints.

The rationale for this study was to explore the extent to which existing definitions reflect recent examples of curriculum co-creation in theory and practice, with respect to this burgeoning field of study. The five influential definitions cited above focus on who is involved (students and staff) and what they are doing (collaborating to develop aspects of the curriculum), with only two emphasising why this work is important. The 2011, 2014 and 2016 definitions developed by Bovill, Cook-Sather and Felten (with colleagues Millard and Moore-Cherry in 2016) highlight approaches to developing aspects of the curriculum, and Ryan and Tilbury (2013) and Dollinger et al. (2018) underscore participatory processes of developing knowledge and wider educational experiences, respectively. Existing definitions do not fully capture the intentionality of creating opportunities for curriculum co-creation or how these collaborations are enacted when sharing decision-making.

## An overview of curriculum co-creation literature

Recent literature was reviewed in a systematic manner to understand the nature of curriculum co-creation principles and practices in higher education, using the Education Resource Information Centre (ERIC) database. The inclusion domain included all conceptualisations, practices and outcomes of all forms of co-created academic experiences and only those examples of co-creation that included both students (undergraduate and/or postgraduate) and staff (academic and/or professional services). The search term ‘co-creat*’ yielded 556 publications from 1 January 2014 to 31 December 2019 including keywords such as ‘co-create’, ‘co-creation’ and ‘co-creating’. Of these, 243 sources were excluded when applying the timeframe inclusion criteria, and 111 further sources were excluded when filtering by ‘higher education’.

Reviewing the titles and abstracts of the 132 sources that met the inclusion criteria, 61 further sources were excluded due to focusing on non-academic higher education experiences, research and/or examples of co-creation between staff in different departments, staff and employers or partners or students with other students. Of the 50 remaining results focusing on co-creation between staff and taught undergraduate or postgraduate students, 34 provided examples of course-level, curricular co-creation and 16 provided theoretical contributions and/or analysed the co-creation of wider learning and teaching initiatives. Additionally, relevant literature found through citations included related topics such as staff-student partnerships and collaborations or participatory curriculum development. The literature provides many rich examples of outcomes from curriculum co-creation (including both benefits and challenges), but here I focus on key themes relating to the nature of co-creative practices: the relationships underpinning co-creation and the ways of working during this process.

### Relationships underpinning curriculum co-creation

Curriculum co-creation (like student/staff partnerships) is distinct from other forms of student engagement—including active learning, student-centred learning and inquiry-based learning—because of its collaborative ethos (Bovill, 2020b; Lubicz-Nawrocka, 2019b; Matthews et al., 2018a; Moore-Cherry, 2019). Co-creation critically involves negotiation and collaboration as opposed to full student control over curricula (Cook-Sather et al., 2014), requiring a positive relationship between students and staff (Bovill, 2020a). This aspect of negotiation is important because staff take ownership over quality assurance and assessment outcomes, but they can create opportunities for students to share decision-making affecting how or what they learn (Boomer, 1992). Building relationships can promote an open dialogue about meaningful teaching and learning, which helps both students and staff to understand their responsibilities and creates a more equal balance of power between them (Boomer, 1992; Lubicz-Nawrocka, 2019b; Moore-Cherry, 2019). Although previous definitions of curriculum co-creation focus on collaboration between students and staff, they do not highlight negotiation which critically recognises constraints and notions of power (Boomer, 1992).

Curriculum co-creation promotes strong working relationships through collaboration not only between students and staff (Blau & Shamir-Inbal, 2018; Kaur et al., 2019; Lubicz-Nawrocka, 2019b) but also between different students through peer learning (Bovill, 2020b; Vaughan et al., 2016). This practice can foster staff and students’ senses of belonging (Cook-Sather et al., 2018a; Masika & Jones, 2016) and community (Blau & Shamir-Inbal, 2018; Lubicz-Nawrocka, 2019a). This literature broadly focuses on valuing students’ views and perspectives, but only limited examples explicitly reference democratic values underpinning co-creational relationships (Bergmark & Westman, 2016; Bovill, 2020a; Lubicz-Nawrocka, 2019b).

### Ways of working that focus on authentic and enjoyable processes of learning and teaching

Curriculum co-creation often presents new ways of working in higher education that can challenge the status quo of academic cultures, hierarchies and norms (Bergmark & Westman, 2016; Bovill et al., 2016). The context-specific, dialogic process underpinning how students and staff share responsibility is now widely recognised (Bovill, 2020b; Dollinger et al., 2018; Healey & Healey, 2018). Co-creation practitioners may engage in creative risk-taking whilst they share power and engage in innovative curriculum design together (Blau & Shamir-Inbal, 2018; Kaur et al., 2019; Lubicz-Nawrocka, 2019a).

Curriculum co-creation can promote authenticity by advancing culturally and personally relevant forms of learning and teaching (Backhouse et al., 2019; Temple Clothier & Matheson, 2019), which is especially relevant in the context of large class sizes and digital education. Students’ deep and active learning is an integral aspect of co-creation of content and pedagogy (Backhouse et al., 2019; Vaughan et al., 2016) as well as assessment (Bovill, 2020b; Doyle et al., 2019). Curriculum co-creation can also help individuals overcome challenges by working collaboratively and resiliently to find authentic solutions that are relevant to their own context (Bergmark & Westman, 2016; Blau & Shamir-Inbal, 2018; Bovill et al., 2016). By fostering more engaging forms of learning and teaching, students and staff tend to find curriculum co-creation processes extremely enjoyable, meaningful and rewarding (Dollinger et al., 2018; Kaur et al., 2019; Lubicz-Nawrocka, 2019a; Temple Clothier & Matheson, 2019). The authentic and rewarding process of curriculum co-creation can motivate the deep engagement of both students and staff to take up these opportunities (Bergmark & Westman, 2016; Lubicz-Nawrocka, 2019b).

## Methodology

### Research question

This research formed part of a larger, doctoral research study to understand the nature of curriculum co-creation principles and practices and how it may advance individuals’ aims for students in higher education. The aspect of the study explored here focuses on the research question: *how do students and staff experience and conceptualise curriculum co-creation?* This research was designed to explore whether previous definitions of curriculum co-creation fully captured staff and students’ understandings of what curriculum co-creation looks and feels like, both in examples from recent literature and in practice in one higher education context.

### Methodological approach

My research question lent itself to qualitative methods of investigation and a heuristic inquiry approach to exploring meaning-making in ways that are exploratory, reflexive, relational and participatory (Nevine, 2019; Punch, 2006). This approach was well suited to help me understand the subjective and nuanced nature of student and staff conceptualisations of curriculum co-creation. Most previous research either focused on principles of co-creation whilst drawing on a range of international examples or they focused on single case study initiatives that highlighted practices within one course, programme or institution. I felt it would be beneficial to identify a diverse variety of curriculum co-creation initiatives within one, discrete higher education context. I selected the Scottish context based on its focus on quality enhancement in learning and teaching. I was not aiming to gather representative data from the sector as a whole but, rather, wanted to focus on in-depth accounts within reasonable resource restrictions.

### Sampling of participants

During my data collection in 2015–2016, there were few examples in Scotland that staff or students intentionally described with the term ‘curriculum co-creation’. Using criterion sampling focused on cases that fit Bovill et al.’s (2011) necessarily broad definition of curriculum co-creation as collaboration between staff and students on aspects of curricula, I used publications, conference presentations and word of mouth to identify 19 staff co-creators at Scottish universities. Whilst trying to avoid oversampling in terms of their subject area, gender or institution, I invited 15 staff to take part in a semi-structured interview. Thirteen of these staff participated and I used snowball sampling to identify 11 students who had co-created curricula with them.

Participants were engaged in 15 curriculum co-creation initiatives across 8 subject areas at the 5 Scottish universities, with several staff involved in more than one form of curriculum co-creation. Participants’ subject areas ranged from medicine and veterinary studies to science (geoscience and biology) and social sciences (politics, sociology, social work and education). In alignment with Bovill et al.’s (2011) definition of curriculum co-creation, the 15 initiatives included at least one aspect—and potentially all aspects—of planning (10 instances), implementation (11 instances) and/or evaluation (7 instances) of students’ learning experience (see Table 1). Co-creating curriculum plans can include development of educational resources for future student cohorts or collaboratively planning and often implementing how later parts of course teaching, assessment or marking will take place. Course co-evaluation involves students and staff identifying strengths and areas for course enhancement, often relating to student engagement. Co-creation in planning, implementation and evaluation is often overlapping, as seen in the literature and in my sample. In an example of evaluation and planning (Initiative 2), staff worked with students who had recently completed a course to identify difficult concepts and work together during a summer project to address them with co-created, multimedia educational resources. Examples of whole-class implementation included co-creating grading criteria (Initiatives 11 and 14), exam questions (Initiative 7) or projects with community partners in service learning and science outreach (Initiatives 4 and 12).

**Table 1:** Participants’ 15 co-creation projects

Initiative number	Co-creation project	University type	Subject type	Co-creation types	Students participating	Staff participating
1	Co-created aspects of a course	Research-intensive	Arts, humanities and social science	Implementation	Whole cohort (earning course credit)	Academic staff
2	Co-created educational resources	Research-intensive	Clinical sciences	Planning & evaluation	Selected student (receiving payment)	Academic staff
3	Staff-supported peer teaching across year groups embedded into courses	Research-intensive	Clinical sciences	Planning & implementation	Whole cohort (earning course credit)	Academic staff
4	Co-created course with community projects	Research-intensive	Science or engineering	Planning & implementation & evaluation	Whole cohort (earning course credit)	Academic staff
5	Co-created aspects of a course including co-development of resources for a new course	Research-intensive	Arts, humanities and social science	Planning & implementation	Whole cohort (earning course credit)	Academic staff
6	Co-created educational resources	Research-intensive	Clinical and veterinary sciences	Planning & evaluation	Selected student (receiving payment)	Academic staff
7	Co-created educational assessment	Research-intensive	Clinical and veterinary sciences	Planning & implementation	Whole cohort (earning course credit)	Academic staff
8	Co-created aspects of a course	Teaching-focused	Science and engineering	Planning & implementation	Whole cohort (earning course credit)	Academic staff
9	Co-created research	Teaching-focused	Science and engineering	Evaluation	Selected students (volunteering and gaining professional development skills)	Academic staff
10	Student learning and teaching consultants	Teaching-focused	Various: arts, humanities and social science/science and engineering	Planning & evaluation	Selected students (volunteering and gaining professional development skills)	Academic staff and academic developer
11	Co-created aspects of a course including marking criteria	Research-intensive	Arts, humanities and social science	Implementation	Whole cohort (earning course credit)	Academic staff
12	Co-created community projects with co-assessment by staff and student	Research-intensive	Arts, humanities and social science	Planning & implementation & evaluation	Whole cohort (earning course credit)	Academic staff
13	Co-created aspects of a course	Research-intensive	Science and engineering	Implementation	Whole cohort (earning course credit)	Academic staff
14	Co-created aspects of a course including marking criteria	Teaching-focused	Arts, humanities and social science	Implementation	Whole cohort (earning course credit)	Academic staff
15	Co-created aspects of a course	Teaching-focused	Arts, humanities and social science	Planning & implementation & evaluation	Selected students (volunteering and gaining professional development skills)	Academic staff and academic developer

### Ethics and data collection

The research received Level 1 ethical clearance from the Moray House School of Education and Sport (University of Edinburgh) Ethics Committee, and the aims of the study and the voluntary nature of participation were made transparent through using participant information sheets and consent forms. In-depth, semi-structured discussions were held with participants who describing their understandings of the higher education curriculum and of curriculum co-creation, the nature of their curriculum co-creation activities and their experiences including collaborative relationships and ways of working. All data collection was audio-recorded and transcribed.

### Data analysis

The extensive qualitative data was analysed whilst drawing on a constructivist grounded theory approach, which is an ‘inductive, iterative, interactive and comparative method geared towards theory construction’ (Thornberg & Charmaz, 2012, p. 41). This data analysis method helped me to draw on existing curriculum co-creation literature to identify core themes that were grounded in my data about participants’ conceptualisations of co-creation of the curriculum. I used the NVivo qualitative data analysis software to facilitate systematic coding and analysis of the extensive qualitative data that consisted of 223,000 words of transcriptions.

I drew on Pillow’s (2003, p. 181) ‘four reflexive strategies—reflexivity as recognition of self; reflexivity as recognition of other; reflexivity as truth; reflexivity as transcendence…’. Therefore, I worked to (1) recognise my background and assumptions; (2) acknowledge different forms of power when working with participants—particularly students—whilst promoting their agency; (3) strive to achieve a sense of validity for my conclusions through constant comparisons across the body of data from different participants; and (4) minimise bias by corroborating my findings with wider literature when presenting my findings.

## Curriculum co-creation themes

Based on my inductive analysis of the data, the findings highlight conceptualisations of curriculum co-creation that focus on three key aspects of (A) developing shared values, (B) enhancing creativity through collaboration and (C) negotiating power for mutual benefit. Each of these themes is explored below.

### Developing shared values

Respondents outlined five values that they viewed as underpinning curriculum co-creation: shared responsibility, reciprocity in learning from each other, mutual respect and care and trust and empathy. Each is described briefly below.

#### Shared responsibility

Both student and staff participants described how they create a new space and ethos for curriculum co-creation to take place where they feel joint ownership over aspects of the curriculum. Staff 1 described the importance of speaking openly to develop shared responsibility during curriculum co-creation that differs from more traditional teaching:

…you have to be very careful not to appear as if you are requiring students to do the work that you should be doing. They have to understand why certain levels of responsibility might be expected of them.

Staff 2 also emphasised that facilitating authentic engagement can motivate students to share responsibility and see it as an opportunity as opposed to a burden, particularly within the context of increasing tuition fees.

Others described sharing responsibility in curriculum co-creation as feeling: ‘It’s not them and us, it’s just *us*’ (Staff 4). Furthermore, Student 11 described the importance of intentionally working towards shared aims:

It was about how everybody would come with some skills or some knowledge and it would all go towards one goal. …I think it’s where you know that you can learn from each other and you can move forward in creating something good for both of you, more than just your own individual use.

The process of developing both individual and collective ownership—in not only learning but also in decision-making affecting teaching—is important in sharing responsibility. Whilst staff and student co-creators take responsibility for different aspects of engagement, their joint ownership often increases their intrinsic motivation to work together.

#### Reciprocity in learning from each other

There are strong overlaps between reciprocity and empathy, and respondents spoke of how co-creation helps them to learn from each other to improve curricula. Student 1 described co-creation as:

…openness on the part of the teacher, and willingness or being receptive on the students’ front. …Obviously the student has to be conscientious, receptive and willing to learn from the expert; but equally the tutor or the professional has to be open to the students’ ideas and willing to have their perceptions changed.

Similarly, Staff 11 argued that curriculum co-creation can develop reciprocity that can overcome traditional, hierarchical relations:

The lecturers didn’t know the answers. It was the first time a lecturer has ever asked them [students] for their view and basically said ‘well how would you do it? …Your solutions are equally as important’.

It can be challenging for some staff to be open to students’ ideas about different ways of learning and teaching, and it can be difficult for some students to engage more actively. However, participants described how they worked to solve complex problems together, since curriculum co-creation advances reciprocity between students and staff who bring different knowledge, expertise and skills that are each valued.

#### Mutual respect and care

Many staff and student co-creators emphasised how respect is both a prerequisite for and an outcome of effective curriculum co-creation, and care is a related value that goes beyond notions of respectful tolerance. Many participants highlighted the responsibility of staff to respect, support and value students’ contributions throughout curriculum co-creation. This may be because staff often set the tone for the learning environment, and students tend to respect their subject-related and teaching-related expertise. However, participants such as Students 3, 6 and 9 described how co-creating curricula makes them feel respected and valued, in sharp contrast with some of their other academic experiences when they felt staff do not provide adequate support or respect their time or individual learning needs.

Staff 3 described respect and care as key values that underpin her curriculum co-creation work:

It was about showing students that we actually really care about teaching. Our students are often first generation to come to university, and it’s great building their enthusiasm. …They feel their work is valued now.

In addition, Student 7 described how her teacher:

…said in the very beginning when we first all got together, ‘we have students who are studying something. They’re a resource, why doesn’t the community use it?’. I think that’s a great way of looking at it, and it teaches us that we have something to offer.

Staff show students respect by recognising that they can make important contributions to curricular decision-making and enhancement, which can help motivate students’ active engagement. For example, Student 11 said:

I felt valued as a student because I wasn’t [feeling like] just one in thousands. I felt that I could make a difference…

The themes of respect and care show how staff attitudes can influence students’ intrinsic motivation and success.

#### Trust and empathy

Many students and staff described developing trust and empathy when curriculum co-creation bridged the academic hierarchies between them. As a foundation, participants highlighted transparency and open communications about expectations, processes and opportunities. Student 3 explained how curriculum co-creation helps students to trust academics and ‘understand the human side of academic staff’, and Student 8 described:

I’ve never had to do anything like it before, but I guess that’s a positive thing… It made me appreciate how hard it must be to be a teacher.

Other participants described how curriculum co-creation facilitates empathy not only on the part of students but also for staff. Staff 10 said:

I think that the more you engage students in activities like this, the more they empathise with the role that academics play. That comes back to bridging the gap between staff and students, bringing the communities closer together. …It gave them [students] that insight into what it is like from the other side… [For staff] it is learning something that you couldn’t have gained without students’ insight.

Learning environments built on trust can support both staff and students to challenge themselves and work collaboratively to overcome challenges in curriculum development. For example, Staff 2 reflected:

A lot of it is about how we take the constraints that we have to live with and work against them to create those communities of trust and genuine learning.

Furthermore, Staff 3 described students’ wider learning:

They gained respect for teaching, and they learnt why decisions are made. They want to know.

When staff include students in curricular decision-making, trust and empathy can help them see each other as ‘human’ and work together to tackle challenges in curriculum co-creation.

### Enhancing creativity through collaboration

Several examples of reciprocity and empathy above show how staff and students welcomed different perspectives whilst working collaboratively and creatively. Respondents described how co-creation can facilitate creativity and innovation in curriculum design since collaborative engagement can enhance curiosity, exploration and connection-making. For example, Student 5 described ‘bouncing ideas off each other’ to advance learning and Staff 2 said:

In terms of creating learning within a social environment, I think that can be a very constructive, imaginative, and creative process and it’s around how we do that collectively…

Many staff spoke about how curriculum co-creation helps them facilitate more vibrant learning environments by drawing on the community’s collective intellectual resources and ideas.

In addition to the collaborative processes of curriculum co-creation, participants highlighted the development of creative experiences and resources. Staff 7 described:

There’s a symbiosis between us and things that are in the ether now that weren’t there before… [it’s] creating learning materials, creating learning experiences: this idea of the whole being more than the sum of its parts. It’s a dialogue between the lecturer and the students.

Student 11 also shared her perspectives on curriculum co-creation:

It’s getting into an environment where you are able to really get your creative side out and trying to see what you can do…

For this student, engaging in curriculum co-creation means being intrinsically motivated to explore opportunities and push traditional boundaries by creatively developing the curriculum in ways that benefit others.

### Negotiating power for mutual benefit

In addition to the shared values, creativity and collaboration that are foundational to curriculum co-creation, participants described how they negotiate power in non-traditional student/teacher relationships. Staff 3 noted that teachers often take the lead initially in curriculum co-creation:

I designed this project so I would always have more power in the sense that I was creating its direction and setting it up against expected outcomes… but there is room for all voices in the discussion.

Similarly, students described how staff should lead in quality assurance based on their subject and teaching expertise, although ‘there is definitely an element for students to come in’ to enhance curriculum development (Student 9).

Participants often discussed how power can shift between staff and students throughout their collaboration. Student 3 emphasised that it takes time to build effective relationships:

…through regular meetings and communications, it did get to that shared platform of partnership. …I think both partners have to step into something neutral and they’ve got to really think about how they’re doing that. There was… a willingness and positive intent that informed how everyone would do it.

Staff and students’ intentionality in curriculum co-creation sets the tone for adopting new ways of working collaboratively. Participants described how students bring different experience and need different levels of support to engage in co-creation, with power dynamics that change based on those engaging and how power ‘oscillates depending on what’s happening… across time and context’ (Staff 1).

By negotiating learning and teaching, co-creators can develop a shared sense of purpose and overcome challenges together. Participants acknowledged challenges such as how it can ‘be a bit daunting because you are letting go of some of the power the teacher would have in the classroom’ (Staff 6) and how ‘sometimes we have to work harder as students’ (Student 6). However, participants including Staff 8 highlighted the enjoyable and rewarding nature of curriculum negotiation:

What was interesting was how much the faculty enjoyed it, in terms of really getting engaged and thinking about the issues... It was a great experience. I hadn’t anticipated how much students would have enjoyed it too. I think it took a certain amount of trust on both of our parts…

Furthermore, Student 8 said:

It was, to be honest, the only course where I felt like I was actually making a difference and had the opportunity to apply knowledge that I had learned.

It is salient that all participants in this study highlighted that curriculum co-creation was a mutually beneficial experience.

## Discussion

The findings show that students and staff engaged in curriculum co-creation conceptualise it as a relational pedagogy in which shared values and strong relationships are foundational, and it is a way of working to negotiate curricula through a creative, reciprocal process that is likely to benefit students and staff alike. Below, I discuss these key themes and demonstrate analytical generalisation by showing how my findings relate to relevant concepts and theories. I then present a new definition that encompasses current conceptualisations of curriculum co-creation.

### A values-based, creative process

In the findings above, the values of shared responsibility, reciprocity in learning from each other, mutual respect and care and trust and empathy are often central to curriculum co-creation. Although the values underpinning co-creation could vary—based on those contributing, the relationships between them, and the contexts within which they work (Bovill, 2020a; Cook-Sather et al., 2014; Mercer-Mapstone et al., 2017)—what is important is that these values are shared. Cook-Sather et al.’s work (2014, p. 1) was influential in emphasising that ‘partnerships are based on respect, reciprocity and shared responsibility between students and faculty’. They suggested that respect is an attitude of openness promoting two-way communication, reciprocity is a way of thinking that advances equity and responsibility is an action of taking ownership (Cook-Sather et al., 2014, pp. 3–5). Participants in my research reinforce the importance of these values and show how they can promote attitudes and actions that demonstrate co-creators’ eagerness to learn from each other’s expertise and diverse perspectives.

Besides Cook-Sather et al.’s (2014) brief mention of reciprocity in their definition of partnership in learning and teaching, values are not explicitly highlighted in established curriculum co-creation definitions. However, examples above from this study and from the work of Bergmark and Westman (2016) and Chappell and Craft (2011) show how developing shared values reflects the intentionality behind the relationships that are cultivated whilst co-creating curricula, including the foundational mindsets that help participants feel included and motivated to contribute actively. Extending others’ previous work concerning shared responsibility, respect and reciprocity I also identified care, trust and empathy as significant shared values in curriculum co-creation. Creativity is another foundational aspect of curriculum co-creation processes explored below.

#### Care

Beyond respect and openness to other views and experiences, participants emphasised care and compassion so that student co-creators ‘feel their work is valued now’ (Staff 3), that they ‘have something to offer’ (Student 7) and that they can ‘make a difference’ (Student 11) through their contributions. This theme of care in curriculum co-creation resonates with others’ findings that teachers’ care and commitment can influence students’ choices and motivations to engage with learning (Bovill, 2020b; Lubicz-Nawrocka & Bunting, 2019; Noddings, 2005). Furthermore, Noddings (2005) highlighted the importance of teachers developing students’ ‘capacity to care’ (p. 18) for their learning and for others, and Matthews et al. (2018b) showed that care can foster ‘more personally transformative outcomes that resonate with broader collective and community goals of higher education as a social good’ (p. 966).

#### Trust and empathy

Shared responsibility, respect, reciprocity and care often support the development of other key values during curriculum co-creation including trust and empathy, which can humanise the higher education experience for all involved (Backhouse et al., 2019; Chappell & Craft, 2011; Lubicz-Nawrocka, 2019b). Whilst there is little research on the role of empathy in higher education, Chappell and Craft (2011, p. 365) show how ‘communal creativity is particularly important to the humanising process and encourages a strong focus on empathy, shared ownership and group identity. …As valuable new ideas emerge from joint embodied thinking and shared struggles, humanising is the process of becoming more humane, an active process of change for the creative group’.

Similarly, participants in my study shared how trust and empathy supported co-creators to engage with non-traditional learning and teaching that could be considered a risk for some when sharing power, being open to different ideas, and dealing with potentially unfamiliar experiences. For staff, risks could involve feeling daunted and uncertain about what students’ input will bring in diversifying curricula and ways of working. For students, risks could mean stepping outside of their comfort zone to engage more actively and learn about both the constraints and possibilities associated with curriculum development. Empathy and trust can help staff and students navigate the aforementioned risks and challenges whilst ‘bridging the gap between staff and students, bringing the communities closer together’ (Staff 10) and helping students ‘understand the human side of academic staff’ (Student 3). Building student/staff relationships based on trust is essential when addressing constraints whilst negotiating curriculum development (Boomer, 1992; Green, 2021) and taking on challenges of working in new ways and taking creative, relational approaches to curriculum development (Bovill, 2020b; Lubicz-Nawrocka, 2019a; Matthews et al., 2018b). However, the importance of trust has not been mentioned in existing definitions of curriculum co-creation.

#### Creativity

Developing shared values, particularly trust and empathy, is key to advancing non-traditional ways of working during curriculum co-creation that often fosters creativity and innovation in curriculum development. This is reinforced by the work of Chappell and Craft (2011), referenced above in relation to how empathy and communal creativity can humanise higher education. Furthermore, curriculum co-creation ‘can be a very constructive, imaginative and creative process’ (Staff 2) with ‘the whole being more than the sum of its parts’ (Staff 7). Although the words ‘co-creation’ and ‘creativity’ share the same etymology, the importance of creativity in curriculum co-creation has been rarely noted in the literature and is absent from current definitions. I previously demonstrated the importance of recognising creativity as a core aspect of co-creation since it develops adaptability and resilience, and it underpins both the creative process of collaborative learning and also the creative projects and resources that are produced as a result (Lubicz-Nawrocka, 2019a).

Wider educational literature describes how negotiated processes of curriculum development can be more inclusive by inviting students to contribute to that creation process (Boomer, 1992; Bron et al., 2016; Green, 2021), and creativity can reframe and enhance current educational practices to engage students who learn in different ways (Gee, 2003). Furthermore, students and staff often take on non-traditional roles and identities whilst co-creating curricula and working in partnership in democratic and egalitarian learning communities (Bergmark & Westman, 2016; Lubicz-Nawrocka, 2019b; Matthews et al., 2018a; Mercer-Mapstone et al., 2018). Therefore, it is important to acknowledge the centrality of creativity within curriculum co-creation processes.

### Staff and students sharing and negotiating decision-making about aspects of curricula, often leading to mutual benefits

The process of students and staff developing shared values, such as those detailed above, underpins how they share decision-making and collaboratively negotiate aspects of curricula. For participants, curriculum co-creation means working together towards shared aims, feeling that ‘we are all in this together’ (Staff 8) and that ‘it’s not them and us, it’s just *us*’ (Staff 4). The process-focused, relational dimension of staff and student collaboration in sharing decision-making is important here, and it is prevalent in the literature that notes the sense of community and belonging that develops during curriculum co-creation (Blau & Shamir-Inbal, 2018; Bovill, 2020b; Cook-Sather et al., 2018a; Kaur et al., 2019; Lubicz-Nawrocka, 2019a; Masika & Jones, 2016).

Co-creators recognised that staff often lead in curriculum development through their subject knowledge, teaching expertise and responsibilities for quality assurance; however, staff create opportunities for students to share responsibility over teaching decisions that affect how or what they are learning. It is clear from the results that staff attitudes and behaviours strongly influence students’ engagement with curriculum co-creation by creating ‘room for all voices in the discussion’ (Staff 3) since ‘they’ve got to really think about how they’re doing that… [with] a willingness and positive intent’ (Student 3). This finding is congruous with research showing how teachers’ care, commitment to engaging and supporting students and open invitations to students to participate have a strong influence on students’ choices and motivations to engage with learning generally (Lubicz-Nawrocka & Bunting, 2019; Noddings, 2005) and with curriculum negotiation in particular (Boomer, 1992; Bovill & Woolmer, 2019).

An important finding is that all students participating in a co-created course emphasised that it was the best course across their entire university degree. Although not all individuals who participate in curriculum co-creation may experience benefits and some face challenges, it is important to recognise the potential for mutual benefit. Indeed, both student and staff participants overwhelmingly described their enjoyment of rewarding experiences that advanced their personal and professional development, which is congruent with the literature (Dickerson et al., 2016; Mercer-Mapstone et al., 2017). For participants, co-creation means learning from each other to ‘move forward in creating something good for both of you’ (Student 11) whilst ‘making a difference’ (Student 8), which is in alignment with literature showing that curriculum co-creation can be transformative (Bergmark & Westman, 2016; Bovill, 2020b; Lubicz-Nawrocka & Bovill, 2021; Ryan & Tilbury, 2013). This is especially significant when recognising that the themes underpinning curriculum co-creation have strong overlaps with notions of excellent teaching and learning (Bovill, 2020b; Dickerson et al., 2016; Lubicz-Nawrocka & Bunting, 2019). It is particularly notable that curriculum co-creation supports students and staff to find authentic solutions that are both relevant to their own context in today’s quickly-changing world (Bergmark & Westman, 2016; Blau & Shamir-Inbal, 2018; Bovill et al., 2016; Lubicz-Nawrocka, 2019a).

### A new definition of curriculum co-creation

It is important that definitions are broad enough to include a wide range of initiatives across different contexts, yet specific enough to be clear. As described when reviewing existing co-creation definitions, these focus broadly on student/staff collaborations in developing aspects of the curriculum (Bovill et al., 2011, 2016) or participatory processes that can increase satisfaction (Dollinger et al., 2018; Ryan & Tilbury, 2013). Although Cook-Sather et al. (2014) highlight several important values underpinning student/staff partnerships, the intentionality of developing shared values is lacking from existing definitions of curriculum co-creation since this is critical for strong working relationships.

Based on key themes that emerged from my findings across the Scottish higher education sector and international literature, I offer a new definition that extends beyond broad notions of student/staff collaborations in curriculum development to capture the intentionally fostered values and dynamics as well as the notably positive outcomes:

*Curriculum co-creation is a relational way of working underpinned by shared responsibility, reciprocity in learning from each other, mutual respect, care, trust, and empathy. This values-based, creative process helps staff and students work together to share and negotiate decision-making about aspects of curricula, which often leads to mutual benefits for learners and teachers.*

Whilst each curriculum co-creation initiative is context-dependent and unique based on the individuals involved (Healey & Healey, 2018), the values that underpin co-creators’ relationships may vary and the outcomes may differ. It is also important not to lose sight of how democratically negotiated curricula can challenge the status quo of academic cultures, structures and processes (Bergmark & Westman, 2016; Boomer, 1992; Bovill, 2020b; Lubicz-Nawrocka, 2019b). However, this definition attempts to specify key dimensions of the relational pedagogy that underpins curriculum co-creation—resonating with Bovill’s recent work (2020a)—since its affective nature has the transformative capacity to humanise educational experiences when students and staff feel, as one participant said: ‘it’s not them and us, it’s just *us*’.

## Concluding thoughts

It is hoped that a new definition of curriculum co-creation will clarify the concept to support practitioners by extending discourse on this process-based, values-led work that can benefit both staff and students. By analysing curriculum co-creation conceptualisations within a single national context with respect to theory and international practice, this study differs from most co-creation research, which focuses either on in-depth analysis at one institution or on selected international case studies. My discussion of each of the themes included in my new definition demonstrates theoretical hybridity by showing how my findings relate to a wide range of established concepts. This research demonstrates (A) concept generalisation showing how findings in my study relate to concepts such as care, trust, empathy and creativity that have not been emphasised widely in the co-creation literature and (B) theoretical generalisation showing how results relate to established definitions. This study may also provide opportunities for provocative generalisability if readers are inspired to think about the possibilities for curriculum co-creation in different contexts.

Although I could only include curriculum co-creation initiatives that were made visible within the sector, there may have been a wider number and variety of co-creation examples of which I was not aware if they were not shared beyond classroom walls. Participants co-creating curricula were not generally representative of the populations of students and staff at Scottish universities since all appeared to be highly engaged and motivated. It is important to note that the vast majority of participants self-selected to engage in curriculum co-creation. The lack of cultural diversity in the selection (or self-selection) of student co-creators has been identified as a challenge across the sector (Mercer-Mapstone et al., 2017), which also appears to be the case in my study. Despite these limitations, this study analyses in-depth experiences and conceptualisations of curriculum co-creation with the aim of facilitating dialogue about upscaling these initiatives to become more prevalent and inclusive. It is important for future research to examine what aspects of support or development are needed to enable both staff and students to engage in curriculum co-creation and to explore further the potential for their mutual benefit.

It is powerful that students and staff in this study highlighted the rewarding nature of curriculum co-creation. Although curriculum co-creation is not without its challenges and it may not be appropriate or feasible in every context, it can humanise teaching and learning by empowering both students and teachers. This is particularly relevant with respect to how the COVID-19 pandemic has provoked new ways of working through increasingly digital and hybrid forms of education. Perhaps we can overcome current challenges by learning from staff and students who embrace creativity and innovation whilst co-creating authentic and meaningful curricula. We have seen how co-creators work towards shared values and aims as they home in on what is important to them in higher education—the mutually beneficial process of staff and students working and learning together.

## References

[CR1] Backhouse S, Taylor D, Armitage JA (2019). Is this mine to keep? Three-dimensional printing enables active, personalized learning in anatomy. Anatomical Sciences Education.

[CR2] Barnett, R., & Coate, K. (2004). *Engaging the curriculum in higher education*. Society for Research into Higher Education & Open University Press.

[CR3] Bergmark U, Westman S (2016). Co-creating curriculum in higher education: Promoting democratic values and a multidimensional view on learning. International Journal for Academic Development.

[CR4] Blau I, Shamir-Inbal T (2018). Digital technologies for promoting “student voice” and co-creating learning experience in an academic course. Instructional Science.

[CR5] Boomer G, Boomer G, Lester NB, Onore CS, Cook J (1992). Negotiating the curriculum. Negotiating the curriculum: Educating for the 21st century.

[CR6] Bovill, C. (2020a). *Co-creating learning and teaching: towards relational pedagogy in higher education*. Critical Publishing.

[CR7] Bovill C (2020). Co-creation in learning and teaching: The case for a whole-class approach in higher education. Higher Education.

[CR8] Bovill C, Cook-Sather A, Felten P, Millard L, Moore-Cherry N (2016). Addressing potential challenges in co-creating learning and teaching: Overcoming resistance, navigating institutional norms and ensuring inclusivity in student–staff partnerships. Higher Education.

[CR9] Bovill C, Cook-Sather A, Felten P (2011). Students as co-creators of teaching approaches, course design, and curricula: Implications for academic developers. International Journal for Academic Development.

[CR10] Bovill C, Woolmer C (2019). How conceptualisations of curriculum in higher education influence student-staff co-creation in and of the curriculum. Higher Education.

[CR11] Breen MP, Littlejohn A (2000). The significance of negotiation.

[CR12] Bron J, Bovill C, Veugelers W (2016). Students experiencing and developing democratic citizenship through curriculum negotiation: The relevance of Garth Boomer's approach. Curriculum Perspectives.

[CR13] Chappell K, Craft A (2011). Creative learning conversations: Producing living dialogic spaces. Educational Research.

[CR14] Cook-Sather, A., Bovill, C., & Felten, P. (2014). *Engaging students as partners in learning and teaching: A guide for faculty*. Jossey-Bass.

[CR15] Cook-Sather A, Des-Ogugua C, Bahti M (2018). Articulating identities and analyzing belonging: A multistep intervention that affirms and informs a diversity of students. Teaching in Higher Education.

[CR16] Cook-Sather, A., Matthews, K. E., Ntem, A., & Leathwick, S. (2018b). What we talk about when we talk about students as partners. *International Journal for Students as Partners, 2*(2), 1–9. 10.15173/ijsap.v2i2.3790

[CR17] Dickerson C, Jarvis J, Stockwell L (2016). Staff-student collaboration: Student learning from working together to enhance educational practice in higher education. Teaching in Higher Education.

[CR18] Dollinger M, Lodge J, Coates H (2018). Co-creation in higher education: Towards a conceptual model. Journal of Marketing for Higher Education.

[CR19] Doyle E, Buckley P, Whelan J (2019). Assessment co-creation: An exploratory analysis of opportunities and challenges based on student and instructor perspectives. Teaching in Higher Education.

[CR20] Fraser S, Bosanquet A (2006). The curriculum? That’s just a unit outline, isn’t it?. Studies in Higher Education.

[CR21] Gee JP (2003). What video games have to teach us about learning and literacy.

[CR22] Green B (2021). Re-negotiating the curriculum?. Curriculum Perspectives.

[CR23] Healey, M., & Healey, R. (2018). ‘It depends’: exploring the context-dependent nature of students as partners practices and policies. *International Journal for Students as Partners, 2*(1), 1–10. 10.15173/ijsap.v2i1.3472

[CR24] Kaur A, Awang-Hashim R, Kaur M (2019). Students’ experiences of co-creating classroom instruction with faculty - a case study in Eastern context. Teaching in Higher Education.

[CR25] Lattuca, L., & Stark, J. (2009). *Shaping the college curriculum: academic plans in context* (2nd ed.). Jossey-Bass.

[CR26] Lubicz-Nawrocka T (2019). Creativity and collaboration: An exploration of empathy, inclusion, and resilience in co-creation of the curriculum. Student Engagement in Higher Education Journal.

[CR27] Lubicz-Nawrocka, T. (2019b). “More than just a student”: how co-creation of the curriculum fosters Third Spaces in ways of working, identity, and impact. *International Journal for Students as Partners, 3*(1), 34–49. 10.15173/ijsap.v3i1.3727

[CR28] Lubicz-Nawrocka, T., & Bovill, C. (2021). Do students experience transformation through co-creating curriculum in higher education? *Teaching in Higher Education*, 1-18. 10.1080/13562517.2021.1928060

[CR29] Lubicz-Nawrocka T, Bunting K (2019). Student perceptions of teaching excellence: An analysis of student-led teaching award nomination data. Teaching in Higher Education.

[CR30] Masika R, Jones J (2016). Building student belonging and engagement: Insights into higher education students’ experiences of participating and learning together. Teaching in Higher Education.

[CR31] Matthews KE, Cook-Sather A, Healey M, Tong VCH, Standen A, Sotiriou M (2018). Connecting learning, teaching, and research through student-staff partnerships: Toward universities as egalitarian learning communities. Shaping Higher Education with Students.

[CR32] Matthews KE, Dwyer A, Hine L, Turner J (2018). Conceptions of students as partners. Higher Education.

[CR33] Mercer-Mapstone, L., Dvorakova, S. L., Matthews, K. E., Abbot, S., Cheng, B., Felten, P., . . . Swaim, K. (2017). A systematic literature review of students as partners in higher education. *International Journal for Students as Partners, 1*(1). 10.15173/ijsap.v1i1.3119

[CR34] Mercer-Mapstone L, Marquis E, McConnell C (2018). The ‘partnership identity’ in higher education: Moving from ‘us’ and ‘them’ to ‘we’ in student-staff partnership. Student Engagement in Higher Education Journal.

[CR35] Moore-Cherry N, Walkington H, Hill J, Dyer S (2019). Pedagogical partnerships, identity building and self-authorship in geography higher education. Handbook for Teaching and Learning in Geography.

[CR36] Nevine, S. (2019). *Universal applications of heuristic inquiry: Bridging research and living experience*. SAGE Publications, Inc.

[CR37] Noddings, N. (2005). *The challenge to care in schools: an alternative approach to education* (Second edition ed.). Teachers College Press.

[CR38] Pillow W (2003). Confession, catharsis, or cure? Rethinking the uses of reflexivity as methodological power in qualitative research. International Journal of Qualitative Studies in Education.

[CR39] Punch, K. (2006). *Developing effective research proposals* (2nd ed.). Sage.

[CR40] Ryan, A., & Tilbury, D. (2013). Flexible pedagogies: new pedagogical ideas. https://www.heacademy.ac.uk/knowledge-hub/flexible-pedagogies-new-pedagogical-ideas

[CR41] Temple Clothier A, Matheson D (2019). Using co-creation as a pedagogic method for the professional development of students undertaking a BA (Hons) in Education Studies. Journal of Further and Higher Education.

[CR42] Thornberg R, Charmaz K (2012). Grounded theory.

[CR43] Vaughan, N., Clampitt, K., Park, N., & Prediger, S. (2016). To teach is to learn twice: the power of a blended peer mentoring approach/student response. *Teaching & Learning Inquiry, 4*(2), 1–17. 10.20343/teachlearninqu.4.2.7

